# Supplementary material for: “It Goes Hand in Hand with Us Trying to Get More Kids to Play” Stakeholder Experiences in a Sport and Active Recreation Voucher Program
Source: Int J Environ Res Public Health. 2023 Feb 24;20(5):4081. doi: 10.3390/ijerph20054081 (PMC10001936; doi:10.3390/ijerph20054081)
Supplement: Supplementary file 1 [file ijerph-20-04081-s001.zip › ijerph-2228585-supplementary.pdf]

Supplementary File S1. Semi-structured interview topic guide

Could you tell me about [Organization name] and your role in this organization?
What has been your role in the implementation of the NSW Government's Active Kids program?
How did you hear about the program?
What motivated you and [Organization name] to register and participate in the NSW Government's Active Kids program?
Can you provide detail regarding the approved activity which children can use their Active Kids voucher for?
Can you provide an approximation to what proportion of kids who registered at [Organization Name] used an Active Kids voucher?
What do you think the NSW government is aiming to achieve through the program?
Tell us about your overall experiences of the Active Kids program over the last 12 months?<br>*What has worked well?*<br>*What are the positives to being a registered Active Kids provider and why?*<br>*What has not worked well?*<br>*Challenges of being a registered Active Kids provider?*
What has been the impact of the Active Kids program on….<br>*Membership – numbers, ages/ nationality, gender and disability inclusion*<br>*Staff and volunteers*<br>*Partnerships*<br>*Resource/Finance*<br>*Marketing and promotion*

Has being involved with the Active Kids program influenced the way your organisation operates?
Has the Active Kids program positively impacted linkage and compliance of your affiliate or underpinning programs/organisations/providers?
What would help [Organization Name] to increase the reach and develop new tailored initiatives.
Will you continue to be part of the Active Kids program over the next 12 months?
Additional Comments
